# Supplementary figures and images for: Diagnostic Accuracy of Ultrasound Guided Percutaneous Pleural Needle Biopsy for Malignant Pleural Mesothelioma
Source: J Clin Med. 2024 Apr 29;13(9):2600. doi: 10.3390/jcm13092600 (PMC11084858; doi:10.3390/jcm13092600)

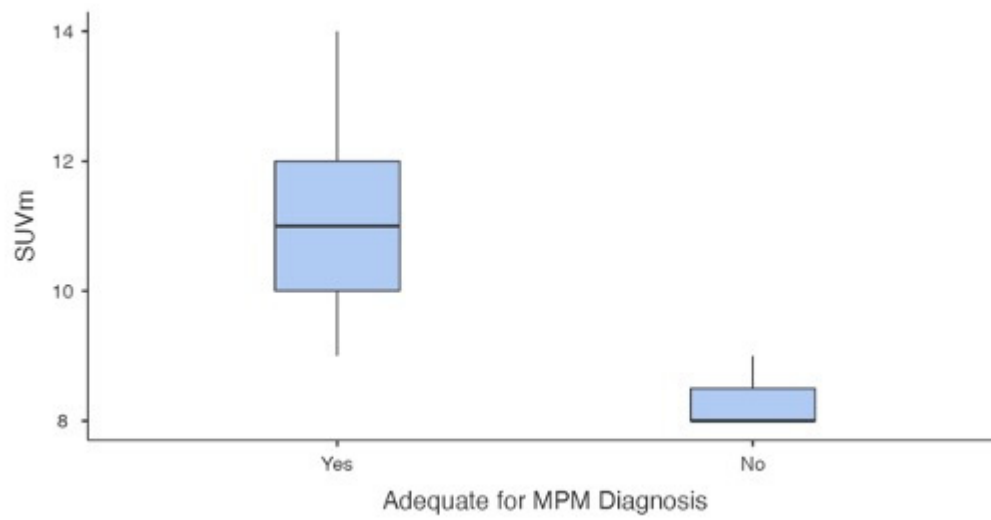

**Figure S1.** Correlation between diagnostic accuracy and 18-FDG-PET-CT avidity value (SUVmax).

Supplement: Supplementary file 1 [file jcm-13-02600-s001.zip › jcm-2919447-supplementary.pdf]
